# Covid-19 pandemic: chronicle of responses and experiences of the infection prevention and control committee at a tertiary hospital in southwest Nigeria

**DOI:** 10.4314/ahs.v21i3.17

**Published:** 2021-09

**Authors:** Akinwumi Ayodeji Akinbodewa, Michael Simidele Odimayo, Olorunfemi Akinbode Ogundele, Tosin Oluwapelumi Ogunleye, Olanrewaju Olayinka Johnson, Oluwakemi Abiola Lamidi, Mathew Akinmurele, Oluwabunmi Motunrayo Oyebade

**Affiliations:** 1 Kidney Care Centre, department of Medicine, University of Medical Sciences Teaching Hospital, Ondo State, Nigeria; 2 Department of Microbial Pathology, University of Medical Sciences Teaching Hospital, Ondo State, Nigeria; 3 Department of Community Medicine, University of Medical Sciences Teaching Hospital, Ondo State, Nigeria; 4 Department of Nursing, University of Medical Sciences Teaching Hospital, Ondo State, Nigeria; 5 Department of Dietetics and Nutrition, University of Medical Sciences Teaching Hospital, Ondo State, Nigeria; 6 Department of Administration, University of Medical Sciences Teaching Hospital, Ondo State, Nigeria

**Keywords:** COVID-19, infection prevention, infection control, Nigeria

## Abstract

Since the advent of 2019-Corona virus Disease (COVID-19) in Nigeria in February 2020, the number of confirmed cases has risen astronomically to over 61,307 cases within 8 months with more than 812 healthcare workers infected and some recorded deaths within their ranks.

Infection prevention and control is a key component in ensuring safety of healthcare workers in the hospital as healthcare-associated infection is one of the most common complications of healthcare management. Unbridled transmission of infection can lead to shortage of healthcare personnel, reduced system efficiency, increased morbidity and mortality among patients and in some instances, total collapse of healthcare delivery services. The Infection Prevention and Control Committee is a recognised group by the Centre for Disease Control and Prevention with their core programmes including drawing up activities, procedures and policies designed to achieve above-stated objectives before, during and after any disease outbreak, especially emerging and re-emerging ones such as the 2019 Coronavirus Disease. In this report, we highlight the roles played by the Infection Prevention and Control Committee of the University of Medical Sciences Teaching Hospital to prevent the spread of COVID-19 within and outside the hospital community and the lessons learned to date.

Healthcare-associated infection is one of the most common complications of healthcare management as it can lead to shortage of healthcare personnel, increased morbidity and mortality among patients and potential shut down of the health system; a situation which is made even more precarious during a pandemic. The 2019 Coronavirus Disease (COVID-19) has become a global pandemic with about 39.2 million people infected in over 213 countries as of October 17, 2020 and more than 1.1 million deaths (case fatality rate: 0.03).^1^

The number of confirmed cases in Nigeria equally continues to rise and currently lies at 61,307 with 1,123 deaths since it was first reported in February in the country with a national case fatality rate of 0.02. The cases are spread across all the thirty six States in Nigeria including Abuja, the Federal Capital Territory.^2^ Currently, Ondo State has recorded 1,654 cases with 36 deaths with a local case fatality rate of 0.02.2 More than 812 healthcare workers have tested positive for SARSCoV-2 with some recorded deaths nationwide.^3^

The University of Medical Sciences Teaching Hospital, Ondo State is a 472-bed tertiary centre established in 2018. Due to its status, it is frequently used as the last point of referral for critical cases from within and outside the State. Top government officials, high profile personalities and foreigners also access care at the hospital. Hence, it is natural that potential cases of COVID-19 will most likely present to the hospital; a history of recent travel to, or return from a COVID-19 prevalent country is one of the case definition criteia for the disease. In this report, we highlight the important roles of the Infection Prevention and Control Committee within and outside the hospital community during the current COVID-19 pandemic, modus operandi and lessons learned so far.

SARS-CoV-2 is a pleomorphic ribonucleic acid virus of the betacoronavirus genus. Its transmission by droplet and physical contact has been established with airborne spread linked only to aerosol generating medical procedures. However, newer evidence (which is largely experimental) has shown that airborne transmission of SARS-CoV-2 may be possible but this position has thrown open further debates considering the fact that its spread by airborne route is not consistent with viral particle size of >5µm and its low viral reproduction number compared to viruses that are ≤5µm in size (2.5 v 18).^4^–^5^

The first step in SARS_CoV-2 infection is the interaction of its Spike (S) proteins with sensitive human cells. Genome encoding then occurs after entering into the cell and facilitates the expression of the genes that encode useful accessory proteins, which advance the adaptation of Coronaviruses to their human host. Virions are then released from the infected cell through exocytosis. The released viruses can proceed to infect kidney cells, liver cells, intestines and T lymphocytes, as well as the lower respiratory tract, where they form the main symptoms and signs.^6^–^7^

On-going efforts to develop a vaccine against SARSCoV-2 by various research groups have reached varying levels of development with twenty five candidate vaccines already at the level of clinical evaluation.^8^ The same can be said for drug development as there are ongoing studies to find the most effective drug(s)/drug combination(s) for its cure.^9^ This unclear status of vaccine and drug development has left the fight against COVID-19 mainly at the level of prevention and control, at least for now.

The currently available options for the management of COVID-19 depends on whether the case is mild, moderate or severe.^10^ The World Health Organization recommends that all cases of COVID-19 should be isolated in order to contain viral transmission. Specifically, mild cases require mainly supportive care and symptomatic treatment (antipyretics, analgesics, adequate nutrition and hydration) while the more severe cases require in-hospital care, antibiotics (where indicated), close monitoring for disease progression, supplemental oxygenation when SPO2 is <90%. Those with Acute Respiratory Distress Syndrome are recommended to receive a trial of continuous positive airway pressure ventilation. The WHO has not recommended the use of choloroquine/hydroxychloroquine, antivirals, immunomodulators and plasma therapy for the treatment or prophylaxis of COVID-19 as existing published literature on these agents are mostly observational in nature with few clinical trials.^10^

## Composition of the Infection Prevention and Control Committee

Infection prevention and control (IPC) is a discipline that aims to prevent or control the spread of infections in healthcare facilities and the community.^11^ The primary goals of an IPC programme are to prevent susceptible patients acquiring disease-causing micro-organisms and to limit the spread of antimicrobial resistant infections. 11–12 The Infection Prevention and Control Committee of the University of Medical Sciences Teaching Hospital was originally constituted in response to an upsurge in the outbreak of new cases of Lassa fever in the last quarter of 2019 in Ondo State with members drawn from various departments (Public Health, Microbiology, Internal Medicine, Medical Laboratory, Nursing, Dietetics, Administration, Public Relations and medical stores) primarily on volunteer basis. This was done to ensure that members were well-motivated personnel who were least occupied by other major responsibilities.

In order to ensure functional efficacy of the Committee members, series of formal training was provided to the team by the Local Government Medical Officer of Health, the Ondo State Epidemiologist and, the Ministry of Health in conjunction with Nigeria Centre for Disease Control (NCDC) under the NiCaDe (capacity development in training of infection prevention and control of health care workers at secondary and tertiary health care facilities) programme.

## UNIMEDTH IPC responses to COVID-19

Our very first step was to conduct a needs assessment at the adult and paediatric Emergency rooms as well as the Obstetrics casualty room to determine their level of preparedness for the COVID-19 pandemic. This was followed by series of specialized training for healthcare workers deployed to these service points which included such modules as the nature of SARS-CoV-2, case definitions for COVID-19, importance of adequate history taking in differentiating between COVID-19 and other respiratory diseases, donning and doffing personal protective equipment and the appropriate communication channel for reporting suspected cases of COVID-19.

## Protocols and policies for managing COVID-19

**Figure 1:**
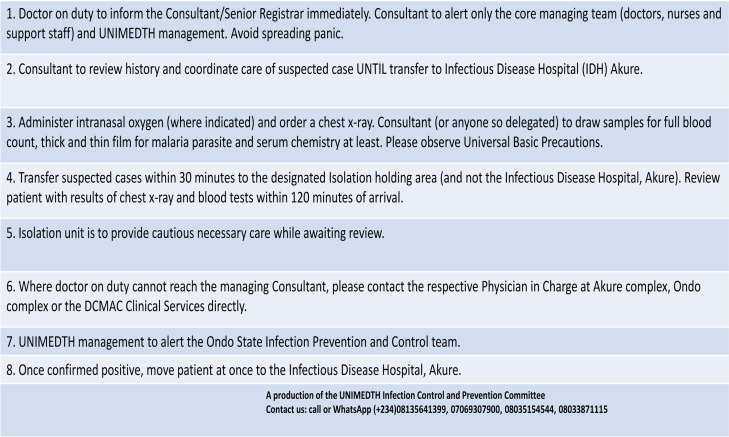
Updated Protocol for managing suspected cases of COVID-19 at University of Medical Sciences Teaching Hospital, Ondo State, Nigeria

A number of protocols have been developed and updated by the Infection Prevention and Control Committee to address management of COVID-19 in the hospital one of which assists medical personnel on duty at the Emergency rooms (figure 1). The key areas of emphasis in the protocol included early involvement of the Consultant and adequate history taking to avoid confusing other respiratory illnesses with COVID-19. Studies have shown that senior physicians painstakingly reach a diagnosis while also requiring fewer investigations.^13^ Their involvement is also associated with better patient outcome while junior doctors have been known to have incomplete doctor-patient communication skills and significantly greater levels of anxiety due to uncertainty. 13–15 These are less desirable qualities required during a pandemic where team stability and tight coordination are key requirements. It should also be noted that during the early period of COVID-19 outbreak in Wuhan, a well-tailored clinical evaluation chart was used to identify suspected cases and rule out potential differentials.^16^ Nevertheless, caution must be applied in strictly relying on clinical evaluation in diagnosing and differentiating between COVID 19 and other respiratory illnesses nowadays as most countries are now officially at the stage of person-to-person transmission and many people (asymptomatic or not) with travel history and contact have not been forthcoming with information on their travel and contact history for fear of discrimination and stigmatization.^17^ Recently, in Nigeria, a number of cases who withheld relevant history have been treated inadvertently by healthcare workers.^18^–^19^

The protocol also emphasized carrying out investigations such as high resolution computerized tomography scan of the chest, full blood count and blood film for malaria parasite among others which could easily rule out common clinical differential diagnosis of COVID-19 in our setting. Employing this strategy has enabled us to triage suspected cases for immediate isolation while awaiting results of Reverse Transcriptase-Polymerase Chain Reaction of nasopharyngeal samples. Among over 40 Persons Under Investigation, 15 have been confirmed positive in our centre while others had diagnoses such as acute severe asthma, community acquired pneumonia, Chronic Obstructive Lung Diseases, non-cardiogenic pulmonary oedema of End Stage Renal Disease etc. This way, emergency needs of patientshave been addressed rather than focus on mounting pressure on the hospital management to “move patient away quickly” to the designated State Infectious Disease Hospital.

A protocol was also developed for the medical laboratory department for prevention of person-to-person transmission among staff. Recently, there have been claims that SARS-CoV-2 attaches to T-lymphocytes in the blood stream thus suggesting that the virus could be blood-borne.^20^ Moreso, there have been questions as to how it is transported from the conjunctiva and other mucosa to the respiratory system.

While we have found these protocols useful in our local setting, they are not without their shortcomings. First is unwillingness of some medical staff to apply the protocols; some have even claimed ignorance of the protocols despite widespread circulation to all the wards, clinics, other service points and social media outlets. Secondly, there were senior nurses and physicians who, out of concern for their personal safety, chose to stay away from making contact with suspected cases thus leaving critical gaps in the execution of the protocols. These attitudes and behaviour have been linked to healthcare workers' discrimination against patients with highly infectious diseases.^21^–^23^

Due to limited financial support, we could not offer free or subsidized High Resolution Computerized Tomography scan to all suspected cases and most importantly, we experienced delay in receiving results of Reverse Transcriptase-Polymerase Chain Reaction tests of suspected cases for SARS-Cov-2, sometimes for up to 4 to 6 days thus exposing many of our patients, relatives and medical staff to undue psychological trauma.

The protocol for managing suspected cases of COVID-19 designed by our IPC team^24^ is an adaptation of the Centre for Disease Control guidelines bearing in mind stark realities of our limited human, capital and technological resources. It is not as elaborate as that used for hospitalised patients under investigation for Ebola viral disease in the United States of America where Patients Under Investigation were isolated in single rooms with fitted private bathrooms, and where there was continuous flow of Personal Protective Equipment for healthcare workers and dedicated medical equipment to care for the sick.^25^ It however aligns to a greater degree with the Centre for Disease Control protocol specifically designed for COVID-19 in terms of practice, Personal Protective Equipment use and reuse but still falls short in some other areas.^26^

## Medical advisories

**Figure 2:**
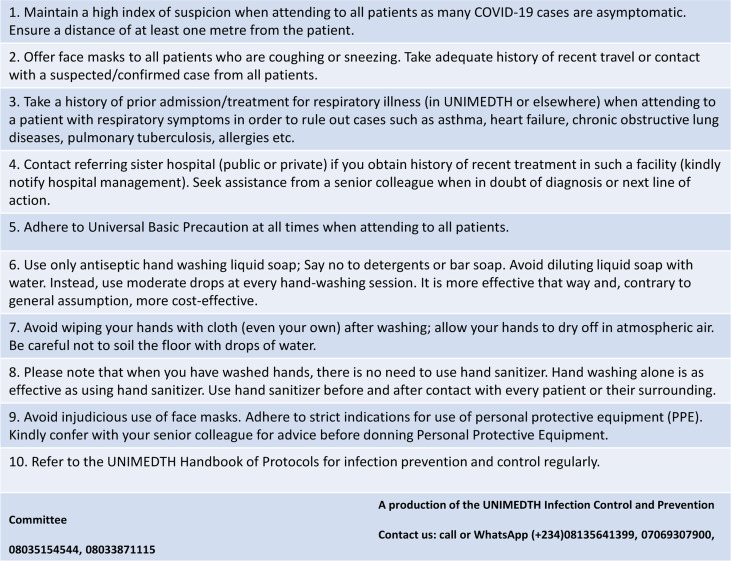
Medical advisory to hospital staff on COVID-19 at University of Medical Sciences Teaching Hospital, Ondo State, Nigeria

In order to ensure continuing medical education on COVID-19 for healthcare personnel, we have published regularly updated medical advisories for them (figure 2). A key element is the combination of cloth facemask and face shield especially with many countries already suggesting unconventional means of optimizing use of face masks and other essential Personal Protective Equipment in the face of dwindling resources.^27^

An advisory was also developed for non-clinical personnel working at the cash collection points. The main purpose of this was to protect them from possible transmission of the virus as paper money and coins could serve as potential vectors for transmission of COVID-19. While this is yet to be proven, a comprehensive review of earlier studies showed that bank notes and coins are capable of transmitting pathogens, even though these studies focused on bacteria, fungi and parasites, probably because most viruses do not survive long outside their host cells.^28^ Whether this could be applied to SARS-CoV-2 is yet to be elucidated as there is currently no available study in that direction but we do know that it survives for hours on different surfaces. In the meantime, the World Health Organization has advised that people should sanitize their hands after handling money.^29^ More importantly, we advised the Accounts department to promote electronic means of payment over cash transaction in order to eliminate or at best, reduce frequent contact with paper money.

## Community engagement and enlightenment

The IPC committee of the University of Medical Sciences Teaching Hospital works with the Disease Surveillance and Notification Officers, Red Cross Society, Environmental Officers and the traditional institutions as part of the Emergency Operation Committee to ensure infection prevention and control in the community. To achieve this during the COVID-19 pandemic, community-sponsored health-related radio Talk Shows were held in the local dialect and English language to reach a wide audience. For emphasis, hospital-sponsored banners bearing the message, “COVID-19 is real: stay home, stay safe” in both English and Yoruba languages were displayed around popular spots in both local governments.

## Local production of Personal Protective Equipment and other hygienic materials

Standard guidelines for healthcare workers on hygienic practices have been released by the World Health Organization. 30 However, similar to what is found in many other developing countries, these have not been easily achievable in our setting when compared to hospitals in developed countries.^31^

For example, hand washing, a key element in infection prevention and control has been difficult to comply with. The reasons for this include low level of awareness of infection control by healthcare staff and poor attitude, lack of time from heavy workload owing to shortage of healthcare work force, as well as lack of wash hand sinks and hygiene products. Secondly, hand disinfection (in between contact with patients) by means of alcohol-based hand sanitizers which takes less time and encourages higher compliance than hand washing is costly and so not always practicable due to limited resources. Other reasons for poor compliance to standard hygienic practices include poor and unsafe working environment, lack of running water and sinks as well as low (and often times irregular) remuneration.^30^ As part of continuing medical education for staff, the IPC committee produced a hospital-sponsored video on donning and doffing of PPE for regular group-viewing. The team was involved in identifying a suitable location for isolating and treating cases of COVID-19, production of locally sourced Personal Protective Equipment, methylated spirit and hand sanitizers.

## Conclusion

The IPC committee's programmes during disease outbreak are essential tools to prevent or reduce infection spread among patients, healthcare workers and the community. The experience garnered so far from serving in the Committee indicates that so much more could be gained if hospitals strengthen their local Infection Prevention and Control Committees.

## Recommendations

The IPC committee should be strengthened in all hospitals at all levels. While the leadership of an IPC team usually should be either a Microbial Pathologist, Community Physician or an Internist, membership should be mainly composed of well-motivated volunteers who are driven by altruism. Hospital managers should be willing to adequately support and fund their IPC committees.

## Limitations

This is a report of a short-spanned, single centre experience.
